# The Epigenetics of Gametes and Early Embryos and Potential Long-Range Consequences in Livestock Species—Filling in the Picture With Epigenomic Analyses

**DOI:** 10.3389/fgene.2021.557934

**Published:** 2021-03-03

**Authors:** Linkai Zhu, Sadie L. Marjani, Zongliang Jiang

**Affiliations:** ^1^AgCenter, School of Animal Sciences, Louisiana State University, Baton Rouge, LA, United States; ^2^Department of Biology, Central Connecticut State University, New Britain, CT, United States

**Keywords:** epigenetics, sperm, oocytes, embryos, cattle, sheep, goats, pigs

## Abstract

The epigenome is dynamic and forged by epigenetic mechanisms, such as DNA methylation, histone modifications, chromatin remodeling, and non-coding RNA species. Increasing lines of evidence support the concept that certain acquired traits are derived from environmental exposure during early embryonic and fetal development, i.e., fetal programming, and can even be “memorized” in the germline as epigenetic information and transmitted to future generations. Advances in technology are now driving the global profiling and precise editing of germline and embryonic epigenomes, thereby improving our understanding of epigenetic regulation and inheritance. These achievements open new avenues for the development of technologies or potential management interventions to counteract adverse conditions or improve performance in livestock species. In this article, we review the epigenetic analyses (DNA methylation, histone modification, chromatin remodeling, and non-coding RNAs) of germ cells and embryos in mammalian livestock species (cattle, sheep, goats, and pigs) and the epigenetic determinants of gamete and embryo viability. We also discuss the effects of parental environmental exposures on the epigenetics of gametes and the early embryo, and evidence for transgenerational inheritance in livestock.

## Introduction

The epigenome carries information encoded by DNA methylation, chromatin configuration, histone modifications, and non-coding RNAs. Drastic epigenome reprogramming occurs during gametogenesis and early embryogenesis, leading to the reset of the epigenetic modifications and the conversion of differentiated gametes into a totipotent embryo. The dynamics of this epigenome reprogramming have been extensively studied in rodent models and humans with fewer studies focused on domestic species. Deficiencies in epigenetic remodeling during this time can cause severe developmental defects, especially in domestic species where assisted reproductive technologies (ARTs) are widely used for research and accelerating genetic selection from genetically superior animals (Sutcliffe et al., 2006). Abnormalities in embryos, fetuses, placentas, and offspring created *in vitro* are thought to be due to improper establishment and/or maintenance of the epigenetic modifications formed during this window (Li et al., 2005; Fernandez-Gonzalez et al., 2010).

Dramatic phenotypic differences have been established and stabilized between livestock breeds by decades and sometimes centuries of selective breeding. These differences include growth rate, presence or absence of horns, muscle characteristics, milk production, heat tolerance, fertility and many others. In some cases, these phenotypic differences can be clearly defined as allelic variations between breeds and are inherited as genetic traits. However, there is phenotypic variation that is not due to underlying sequence differences. In cattle, for example, 32–80% (depending on the trait) of the additive genetic variance can be attributed to genetic variation (e.g., single nucleotide polymorphisms, substitutions) (Haile-Mariam et al., 2013). Thus, there is a missing heritability component (Yang et al., 2010). Furthermore, the interaction of the genotype with environmentally susceptible epigenetic modifications contributes to phenotypic variability and is important to analyze. In many species, there is evidence that environmentally induced epigenetic modifications can persist in future generations serving as a “memory” of past experiences (Daxinger and Whitelaw, 2012; Heard and Martienssen, 2014). Many correlative studies in animal models, such as rats, mice, and even in humans, have suggested various epigenetic factors (e.g., DNA methylation, histone modification, and small RNAs) in germ cells may carry these memories, reviewed in Daxinger and Whitelaw (2012), Heard and Martienssen (2014), Chen et al. (2016b), and Zhang et al. (2019). Numerous studies have looked at the effects of parental environmental exposures on livestock development and production. However, a more limited number of studies in domestic animals have examined the epigenetic mechanisms/modifications responsible, and even fewer have sought to identify transgenerational inheritance.

The development of epigenomic technologies is facilitating the global profiling of germline and embryonic epigenomes, and rapidly improving our understanding of epigenetic regulation and inheritance in a number of species. These achievements provide new areas for the development of promising technologies, or potential management modifications that can neutralize adverse conditions or even improve the performance of livestock. In this review, we outline the current epigenetic characterization of germ cells and embryos in livestock species. We avoid extensive discussion of the epigenetic analysis in model organisms, such as rodents, which have been comprehensively reviewed elsewhere (Smith and Meissner, 2013; Lee et al., 2014; Xu and Xie, 2018; Greenberg and Bourc'his, 2019; Wen and Tang, 2019). We will also discuss studies in livestock species that analyze parental, environmentally induced epigenetics in gametes, the embryo and fetus, and evidence of transgenerational inheritance.

## Epigenetic Reprogramming During Early Embryo Development in Livestock Species

### DNA Cytosine Methylation

5-methylcytocine methylation (5mC) is an important epigenetic modification. It plays essential roles in mammalian development as it is crucial in regulating gene expression, genomic imprinting, silencing of repetitive DNA, differentiation, and X chromosome inactivation (Li et al., 1993; Bird, 2002; Jaenisch and Bird, 2003; Hackett et al., 2013). 5mC can conceal gene regulatory regions and recruit transcriptional repressors and/or chromatin modifiers/remodelers, and therefore is mostly involved in gene silencing (Schultz et al., 2015). However, 5mC has been implicated in transactivation if distributed within the gene body as seen in bovine oocytes and placental tissues (Schroeder et al., 2015). TET enzymes can modify 5mC to 5hmC, 5fC, 5caC, which can be considered as epigenetic modifications in their own right (Inoue and Zhang, 2011; Inoue et al., 2011; Wossidlo et al., 2011). Full characterization of these modifications in early embryos is lacking in livestock species.

DNA methylation is relatively stable in differentiated somatic cells, but highly dynamic during the development of primordial germ cell (PGCs) and preimplantation embryos when global DNA methylation patterns are reprogrammed (Saadeh and Schulz, 2014). In the mammalian embryos studied to date, the first reprogramming event takes place post-fertilization and involves widespread and swift demethylation of the paternal genome, followed by a progressive drop in global DNA methylation of the maternal genome as cleavage progresses (Smith et al., 2012, 2014; Guo et al., 2014; Gao et al., 2017; Jiang et al., 2018; Duan et al., 2019). This nadir is followed by global *de novo* methylation (Mayer et al., 2000; Oswald et al., 2000; Santos et al., 2002; Smith et al., 2012; Gao et al., 2017; Jiang et al., 2018; Duan et al., 2019). The second wave of mammalian DNA methylation reprogramming takes place in PGCs where global demethylation and erasure occurs and imprints are formed based on the sex of the fetus (Popp et al., 2010; Guibert et al., 2012; Hackett et al., 2013; Hill et al., 2018).

Most of what is known about the dynamics of 5mC methylation during preimplantation development in domestic species was revealed by immunostaining (Dean et al., 2001; Beaujean et al., 2004; Park et al., 2007; Deshmukh et al., 2011; Dobbs et al., 2013). While immunostaining provides important overall methylation dynamics, it does not provide specific sequence information of the methylated/de-methylated regions. The more sequence-based approach of a DNA methylation microarray was used to analyzed blastocyst stage embryos in porcine (Bonk et al., 2008) and bovine (Salilew-Wondim et al., 2015, 2018; Ispada et al., 2018). Although considerably more specific than immunostaining, microarrays are limited by the finite number of probes used in their construction and do not provide high-level single-nucleotide resolution of methylation status. Numerous studies have also been performed to evaluate the methylation level of selected candidate genes and regulatory regions in bovine and porcine oocytes and embryos (Gebert et al., 2009; Niemann et al., 2010; Heinzmann et al., 2011; Dobbs et al., 2013; Zhao et al., 2013; O'Doherty et al., 2015; Mattern et al., 2016; Urrego et al., 2017).

Using reduced representative bisulfite sequencing (RRBS), we were the first to report methylome dynamics at single-base resolution in bovine *in vivo* preimplantation embryos (Jiang et al., 2018). It has also been used to assess methylation patterns in many bovine tissues, including the uterus and testes (Zhou et al., 2016). RRBS preferentially selects CpG-rich regions, such as CpG islands, while CpG shores are usually under-represented (Doherty and Couldrey, 2014). These shore regions are known to play important roles in tissue differentiation (Doi et al., 2009). Recently, the development of single-cell whole genome bisulfite sequencing (WGBS) allowed for the reliable and affordable revelation of potentially all CpG sites in a single oocyte or embryo (Smallwood et al., 2014). It also allows for interrogation of 5mC in non-CpG contexts, which are preferentially enriched in oocytes, embryos and stem cells (Lister et al., 2009; Tomizawa et al., 2011) and not clearly mapped in many livestock species. We adopted the WGBS method to further characterize stage-specific genome-wide DNA methylation in bovine sperm, immature oocytes, oocytes matured *in vivo* and *in vitro*, as well as *in vivo* developed single embryos at the 2-, 4-, 8-, and 16-cell stages (Duan et al., 2019). Both studies indicated that the major wave of genome-wide demethylation was completed by the 8-cell stage in bovine embryos. Sequencing-based analyses have profiled the demethylation during preimplantation development in mice (Smallwood et al., 2011; Smith et al., 2012), and primates (Gao et al., 2017) including humans (Guo et al., 2014; Smith et al., 2014). The timing of the major wave of genome-wide demethylation differs from what was observed in bovine embryos, for example, the most marked demethylation occurred at the zygote stage in mice (Smith et al., 2012), at the 2-cell stage in rhesus monkeys (Gao et al., 2017), and at the 4-cell stage in humans (Smith et al., 2012; Guo et al., 2014).

Our analysis also found that sperm and oocytes were differentially methylated in numerous regions (DMRs), which were primarily intergenic, suggesting that these non-coding regions may play important roles in gamete specification. DMRs were also identified between *in vivo* and *in vitro* matured oocytes, reinforcing environmental effects on epigenetic modifications (further discussion of this later). Overall, these characterizations are critical to understanding the epigenetic reprogramming and regulation that occurs during normal, bovine embryonic development *in vivo*, and to providing insight into the epigenetic alterations that occur during *in vitro* maturation (IVM) of oocytes and culture (IVC) of embryos after *in vitro* fertilization (IVF). Importantly, bovine embryos, which are more like human embryos than mouse embryos are in terms of gene expression profiles and developmental timing (Jiang et al., 2014), can serve as a great model for understanding early human development, especially since human *in vivo* embryos are not available for research.

### DNA N^6^-Adenine Methylation

It was widely accepted that 5mC was the only form of DNA methylation in mammalian genomes and that the other modifications were absent, such as N^6^-adenine methylation (N^6^-mA), which is predominantly found in prokaryotes and a limited number of eukaryotes (Heyn and Esteller, 2015). The role N^6^-mA plays in gene regulation and epigenetic remodeling remains essentially uncharted. With the development of next generation sequencing technologies, N^6^-mA was found to be present in several eukaryotes, including *C. elegans* (Greer et al., 2015), green algae (Fu et al., 2015), and Drosophila (Zhang et al., 2015). With the advent of more sensitive detection techniques, N^6^-mA has been more recently identified in *Xenopus laevis* (Koziol et al., 2016), and mammals, i.e., in mouse kidney (Koziol et al., 2016), and embryonic stem cells (ECSs) (Wu T. P. et al., 2016), porcine gametes and embryos (Liu J. et al., 2016), human glioblastoma (Xie et al., 2018), and mouse trophoblast lineages (Li et al., 2020). In pigs, mass spectrometry analysis showed that the N^6^-mA/A ratio in oocytes (0.09%) was ~6 times higher than that in sperm. This ratio rose to ~0.17% from the four-cell to the morula stage and then decreased to 0.05% at the blastocyst stage. However, only a low level of N^6^-mA was observed in genomic DNA of various adult porcine tissues. These results were also confirmed by immunostaining, which further supports the presence of N^6^-mA in porcine early embryos (Liu J. et al., 2016). These findings defy the prevalent theory of 5mC as the only form of DNA methylation in the mammalian genome and suggest that N^6^-mA is conserved and may be important during early development and differentiation (Li et al., 2020). In addition, N^6^-mA in mammals appears to be a repressor of gene expression, that unlike 5mC, is independent of CpG islands and thus would have increased sequence flexibility. The specificity and dynamics of N^6^-mA establishment and removal will be crucial for understanding its role in gametes and embryos.

### Chromatin Remodeling

Accessible chromatin delineates regulatory sequences, such as promoters, enhancers, and locus-control regions. Early mammalian embryos experience extensive chromatin remodeling and proper regulation of chromatin state is essential for transcription and preimplantation development (Burton and Torres-Padilla, 2014). However, it has been difficult to explore global chromatin landscape and its dynamics in gametes and early embryos due to the amount of DNA typically required for such analyses. Recently, low input or single-cell assays to profile chromatin remodeling have been developed [DNase-seq, an assay for transposase-accessible chromatin (ATAC-seq), nucleosome occupancy and methylome analysis (NOMe-seq), and 5C-seq or Hi-C] reviewed by Xu and Xie (2018).

These methods have interrogated chromatin configuration from different angles in gametes and early embryos. The dynamics of chromatin accessibility in gametes and early embryos in mice and humans have been extensively analyzed (Lu et al., 2016; Wu J. et al., 2016; Guo et al., 2017; Inoue et al., 2017; Jachowicz et al., 2017; Jung et al., 2017; Wu et al., 2018; Liu L. et al., 2019). Unsurprisingly, these studies have revealed highly active chromatin landscapes during early development. As one would expect, generally, chromatin was more accessible in embryos after embryonic genome activation (EGA). Interestingly, Guo et al. (2017) reported a more oscillating chromatin state, with chromatin accessibility increasing from gametes to zygotes, followed by a decrease after the late zygote stage, before increasing again in the four-cell embryo. Additionally, by integrating chromatin accessibility with the transcriptome in early embryos, a regulatory network was constructed, and this identified the transcription factors associated with preimplantation development and lineage specification (Wu J. et al., 2016).

Distinct chromatin organizations in gametes and preimplantation embryos have been revealed using the Hi-C approach (Battulin et al., 2015; Du et al., 2017; Flyamer et al., 2017; Jung et al., 2017; Ke et al., 2017). Topologically Associating Domains (TADs) and chromatin compartments in sperm appear to be largely like those in other mammalian cells, such as embryonic stem cells or somatic cells (Battulin et al., 2015; Jung et al., 2017). In oocytes, TADs are present in GV oocytes, but are not observed in MII oocytes (Ke et al., 2017), which makes sense given the transcriptional inactivity at this stage. The re-establishment of TADs after fertilization is slower compared with that observed in somatic cells after division and coincides with EGA (Du et al., 2017; Ke et al., 2017). Allele-specific chromatin architecture was also observed during mammalian embryogenesis. In zygotes, chromatin compartments appear to be absent or faint in the maternal pronucleus, but are evident in the paternal genome (Du et al., 2017; Flyamer et al., 2017; Ke et al., 2017). The 3D chromatin structure across consecutive stages of mouse somatic cell nuclear transfer (SCNT) embryos was also examined using a low-input Hi-C (Chen et al., 2020). This work identified defects in the cloned embryos, specifically stronger TAD boundaries and abnormal interactions between super-enhancers and promoters. Importantly, this research sheds even more light on what is required for successful nuclear reprogramming during SCNT (Chen et al., 2020).

Integrating these datasets from mouse and human gametes and embryos provides a widespread view of the chromatin configuration changes during early embryogenesis. However, the chromatin reorganization in livestock gametes and embryos remains largely unknown. Following our recent efforts to characterize the DNA methylomes of bovine early embryos (Jiang et al., 2018; Duan et al., 2019), we profiled the accessible chromatin in bovine oocytes and early embryos using ATAC-seq (Ming et al., 2020). We generated a high-resolution map of accessible chromatin in bovine oocytes and embryos at the 2-, 4-, 8-cell, morula, blastocyst and elongating stages. We identified distinct gene network programs and transcription factors that differ between *in vivo* and *in vitro* derived blastocysts, which may serve as biomarkers of embryo viability. We also performed an integrative analysis of the transcriptome, DNA methylome and chromatin dynamics and exposed the essential components of the regulatory network controlling bovine early embryonic development. The comprehensive dataset we established will further the understanding of the epigenetic reprogramming that takes place during early bovine embryogenesis. Another characterization of the accessible chromatin in early bovine embryos using the same ATAC-seq approach was recently published (Halstead et al., 2020). Similar to the findings in humans and mice (Lu et al., 2016; Wu J. et al., 2016; Wu et al., 2018), both studies suggest that cattle embryos experience progressive chromatin accessibility during cleavage, which is consistent with the transcriptional activation of the embryonic genome. Interestingly, Halstead et al. (2020) identified a conserved set of maternal factors in mice, cattle and humans that were involved in regulating chromatin remodeling prior to EGA. They also found that the open chromatin regions set during EGA were enriched for homeobox motifs. Overall, open chromatin patterns had significant similarities between cattle and human embryos, providing further evidence that cattle embryos are a good model for human preimplantation development.

### Histone Modifications

Histone modifications are essential for regulating gene expression. Post-translational modifications (PTMs) of histone tails can directly alter the spatial arrangement of nucleosomes on the DNA and the higher-order chromatin structure. Importantly, they can also affect the recruitment of other proteins and complexes onto the chromatin (Kouzarides, 2007). Therefore, the accessibility of chromatin to transcription factors and the subsequent gene expression pattern can be controlled by histone PTMs (Venkatesh and Workman, 2015). Interestingly, histone PTMs can be passed to progeny through gametes (Skvortsova et al., 2018). Conventionally, chromatin immunoprecipitation with high-throughput sequencing (ChIP-seq), which is widely used to survey histone PTMs, requires millions of cells to get high-quality libraries. Modification and improvement of the ChIP-seq methodology and the analysis pipeline [CUT&RUN (Skene and Henikoff, 2017)] has made it possible to retrieve high quality and reproducible data from single cells, reviewed in Xu and Xie (2018). Therefore, it is now feasible to reveal the genome-wide distribution of histone PTMs in gametes and preimplantation embryos.

The reprogramming of histone modifications during early embryo development has been well-established in mice (Dahl et al., 2016; Liu X. et al., 2016; Zhang B. et al., 2016; Zheng et al., 2016; Inoue et al., 2017; Hanna et al., 2018). Both H3K4me3 and H3K27me3 display widespread distal domains in mouse oocytes, which can be inherited in early embryos and regulate zygotic gene expression (Dahl et al., 2016; Zhang B. et al., 2016; Zheng et al., 2016; Inoue et al., 2017). During gametogenesis, distinct histone modifications are established in females and males (Zheng et al., 2016). After fertilization, maternal H3K4me3 from the oocyte is inherited by early embryos, before it is removed upon the start of EGA at the late two-cell stage. By contrast, sperm H3K4me3 and H3K27me3 are quickly removed after fertilization. This is then followed by re-establishment of low-level broad domains across the genome (Zhang B. et al., 2016). Most recently, the remodeling of histone modifications in human early embryo development was reported (Xia et al., 2019). Unlike what is observed in the mouse, the activating mark, H3K4me3, occurs as strong peaks at promoters in human GV oocytes. After fertilization, zygotes displayed widespread H3K4me3 patterns (Xia et al., 2019).

Dynamic changes in histone modifications in gametes and embryos have been widely reported using immunofluorescence in different livestock species, including bovine and porcine (Lepikhov et al., 2008; Ross et al., 2008; Canovas et al., 2012; Herrmann et al., 2013; Diao et al., 2014; Huang et al., 2015; Xie et al., 2016; Chung et al., 2017; Liu et al., 2018). Recently, Org et al. (2019) published the first genome-wide localization of histone H3K4me3 and H3K27me3 in the inner cell mass (ICM) and trophectoderm (TE) of bovine blastocysts. By linking histone PTM profiling and gene expression, this study revealed similar levels of H3K4me3 and H3K27me3 in both up- and down-regulated genes, respectively, in the ICM. In the TE, however, higher levels of H3K4me3 around promoter regions and lower levels of H3K27me3 across the whole gene region were observed in upregulated genes. The authors suggested that together these two histone modifications exert proactive epigenetic regulation in the TE, but not in the ICM (Org et al., 2019). However, it is important to note that locus specific localization of histone modifications has not been investigated across preimplantation embryo development in livestock species to date.

During epigenetic remodeling of bovine embryos, the enzymes that are responsible for the methylation of H3K9me2, H3K9me3, H3K4me2, H3K4me3, and H3K27me3 are known; they include EHMT1/2, SUV39H1/H2, SETDB1, EZH2, and SMYD3 (McGraw et al., 2007; Ross et al., 2008; Golding et al., 2015; Bai et al., 2016; Zhang et al., 2016b). The expression of enzymes responsible for removal of the methylation from H3K4, H3K9, H3K27 were also characterized in bovine early embryos (Glanzner et al., 2018). KDM6B (JMJD3) is involved in the erasure of H3K27me3 during embryo cleavage. Knockdown of *KDM6B* in bovine oocytes resulted in compromised EGA and reduced development to the blastocyst stage (Canovas et al., 2012; Chung et al., 2017). Likewise, knockdown of *SMYD3*, a H3K4 methyltransferase, in bovine *in vitro* matured oocytes negatively impacted embryo development at the 8-cell stage and beyond and resulted in decreased *NANOG* expression in oocytes, but increased expression in early embryos (Bai et al., 2016).

In porcine embryos, H3K4me2/3 is actively demethylated to the monomethylated form (H3K4me1) from 4-cell to blastocyst stage by lysine-specific histone demethylase 5B (KDM5B, also known as JARID1B or PLU-1), whose expression is elevated during this time frame. Demethylation of H3K4me3 is suggested to be important in maintaining proper H3K4me3 (permissive)/H3K27me3 (repressive) ratio during porcine blastocyst formation (Huang et al., 2015), which is consistent with select genes (e.g., members of homeobox family, etc.) being silenced for proper lineage specification during differentiation (Bernstein et al., 2006). The elaborate balance between H3K4me3 and H3K27me3 is also controlled by KDM6B, which is a H3K27me3 demethylase. *KDM6B* knockout alters gene expression at the 8-cell stage and hampers bovine blastocyst formation (Chung et al., 2017).

### Non-coding RNAs

As sequencing technologies advance, we learn that up to 90% of the eukaryotic genome is transcribed to some extent, while messenger RNAs (mRNAs), which are protein-coding, only account for 1–2% of the total RNA population (Ponting and Belgard, 2010). The remaining “non-coding” RNAs can be classified into “housekeeping” RNAs (e.g., ribosomal RNA, rRNA; transfer RNA, tRNA; small nuclear RNA, snRNA; small nucleolar RNA, snoRNA), and “regulatory” RNAs (e.g., small non-coding RNA, sncRNA; long non-coding RNA, lncRNAs) that are involved in modulating gene expression (Kim and Sung, 2012).

In mammals, the “regulatory” ncRNAs have been found to be actively involved in gametogenesis and early embryo development. PIWI-interacting RNAs (piRNAs) and associated PIWI proteins are rarely found in somatic cells, while they are enriched in male germ cells and comprise the majority of sncRNAs present during spermatogenesis in mice (Deng and Lin, 2002; Kuramochi-Miyagawa et al., 2004). It has been shown that piRNAs are indispensable for spermatogenesis and fertility in mice (Fu and Wang, 2014) probably through their role in the repression of transposons (Carmell et al., 2007), where piRNAs facilitate the *de novo* methylation of transposon-encoding genes, therefore preventing their accumulation (Kuramochi-Miyagawa et al., 2008; De Fazio et al., 2011; Siomi et al., 2011).

It has also been established in mice that besides piRNA populations, miRNAs and siRNAs are also present during spermatogenesis and show stage-specific transcription (Hayashi et al., 2008; Song et al., 2011). Nonetheless, our understanding of miRNA and siRNA functionality of germ cells is rather limited compared to what is known about piRNAs. In mice, disruption of sncRNA biogenesis is associated with defective primordial germ cell proliferation, meiotic progression, spermatid condensation/elongation, and elimination of spermatocytes (Maatouk et al., 2008; Romero et al., 2011; Song et al., 2011; Greenlee et al., 2012; Wu et al., 2012; Zimmermann et al., 2014; Modzelewski et al., 2015). Other roles of sncRNAs in murine spermatogenesis, e.g., heterochromatin formation, transcriptional silencing and DNA damage repair, have been reviewed elsewhere (Hilz et al., 2016). Compared with their role in males, miRNAs and piRNAs seem to be non-essential in later stages of oogenesis and in early embryos, at least in mice (Suh et al., 2010; Hilz et al., 2016). Recent studies suggest that this aspect of the functionality of sncRNAs in mice might be the exception in mammals. For example, studies show that PIWI proteins and piRNAs are present in human, macaque and bovine ovaries, indicating their role of transposon repression in oocytes (Roovers et al., 2015). Of note, PIWIL3, one of the four PIWI proteins in most eutherian mammals, is only found in oocytes and not in the testis in bovine, while this copy is completely lost in mice (Roovers et al., 2015). Thus, it appears that sncRNA functions in gametogenesis diverged during evolution.

Our understanding of ncRNAs during gametogenesis and embryo development in livestock species mainly comes from quantitative RT-PCR analyses (Tesfaye et al., 2009; Tscherner et al., 2014; Gilchrist et al., 2016; Berg and Pfeffer, 2018). However, comprehensive profiling of ncRNAs has been conducted in bovine (Gilchrist et al., 2016; Pasquariello et al., 2017; Cuthbert et al., 2019), porcine (Yang et al., 2012; Zhong et al., 2018), and caprine (Deng et al., 2018; Ling et al., 2019) with high-throughput sequencing-based technology. In cattle, miRNA abundance is elevated at 8-cell stage when the major EGA occurs. Of note, the upregulated miRNAs were predicted to target genes involved in cell development, cell division, Wnt signaling and pluripotency, etc. (Cuthbert et al., 2019). In contrast, piRNAs were found in bovine oocytes and blastocysts, but not in 8-cell stage embryos. The shift of sncRNA abundance at 8-cell stage alludes to an important role in activating the bovine embryonic genome (Cuthbert et al., 2019). In porcine, lncRNAs have been involved in oocyte maturation, transcriptional regulation of EGA, first lineage segregation and somatic reprogramming to pluripotency (Yang et al., 2012; Zhong et al., 2018). Interestingly, many of the sncRNAs are mapped to annotated repetitive elements (e.g., SINEs and LINEs) in the pig genome, indicating the regulatory function of these elements during early embryo development (Yang et al., 2012). In goats, 5,160 differentially expressed lncRNAs were identified across developmental stages; the extensive association of lncRNA target genes with other key regulatory genes indicates that lncRNAs are indispensable in embryonic development (Ling et al., 2019). However, the functional characterization of sncRNAs during early embryo development and gametogenesis is still limited in livestock species, so how sncRNAs contribute to the overall epigenetic regulation that takes place during this time is unclear.

Overall, epigenome reprogramming is extensively characterized in the mouse, and most recently, in humans. Recent epigenomic studies building on advances in ultra-low input chromatin profiling methods will help to integrate the DNA methylome, chromatin states, histone modifications and non-coding RNAs during early embryo development in livestock. As discussed, research in these areas is emerging on a large scale. It would be very interesting to combine all available datasets to determine the conserved and divergent epigenome reprogramming programs during early embryo development across different mammalian species. Most importantly, it is critical to determine how the different epigenetic mechanisms regulate the transcriptional program, and how different epigenomic reprogramming events interact with each other to secure successful development.

## Epigenetic Determinants of Gamete and Embryo Viability

### Epigenetics and Oocyte Viability

Success in *in vitro* embryo production (IVP) relies on successful oocyte collection and optimal oocyte quality (Baruselli et al., 2012). However, manipulation of oocytes by superovulation, IVM, oocyte cryopreservation, etc. influences oocyte competency, largely through the introduction of epigenetic abnormalities. Additionally, environmental stressors can reduce oocyte competence even further. The effects of aging on oocyte quality and associated epigenetic changes have been documented and extensively reviewed in humans (Ge et al., 2015). There is growing interest to identify the epigenetic signatures of gametes, and investigate the key molecular drivers that are perturbed at susceptibility loci leading to aberrant gametes in livestock.

Superovulation and oocyte pick up (OPU) have been widely used in bovine IVP programs to increase the number of oocytes from elite animals for assisted reproduction. As observed in human and mouse studies, superovulation can induce aberrant epigenetic profiles and alter gene expression in oocytes and embryos (Khoueiry et al., 2008; Fauque, 2013). In cattle, oocytes retrieved with or without stimulation showed significantly different expression of genes regulating the cell cycle; overall, more than 50% of the genes studied were upregulated after gonadotropin treatment (Chu et al., 2012). Differential DNA methylation of imprinted loci was also detected in oocytes collected from women and mice after superovulation (Shi and Haaf, 2002; Sato et al., 2007). Limited studies have been carried out on the epigenetic landscape of oocytes retrieved from hormonally primed cows. In one study, divergent DNA methylation patterns were found only in satellite sequences, but not in developmentally important, non-imprinted genes (e.g., *SLC2A1, PRDX1, ZAR*) after FSH and IGF1 treatment (Diederich et al., 2012). To determine the ultimate effect of exogenous hormone on oocyte competence and embryo quality, comprehensive epigenomic studies are needed; however, this is limited by the ability to collect enough naturally ovulated oocytes to serve as proper controls.

Oocyte maturation is relatively efficient *in vitro*, especially in cattle and swine, and is now widely used to generate source material for ARTs and gene editing both for agricultural and basic research applications. However, it is well-established that *in vivo* derived oocytes are much more developmentally competent and capable of normal development at higher rates than those *in vitro* matured (Leibfried-Rutledge et al., 1987; Reik and Maher, 1997; Rizos et al., 2002). In cattle, genome-wide methylation patterns during oocyte maturation *in vivo* and *vitro* have been comprehensively investigated (Jiang et al., 2018; Duan et al., 2019). Global DNA methylation of oocytes appears to be stable during the *in vitro* maturation process; *in vitro* maturation maintained GV oocyte-levels of methylation. Whereas, *in vivo* maturation increased DNA methylation levels in both RRBS and WGBS studies (Jiang et al., 2018; Duan et al., 2019). Interestingly, a significant increase in DNA methylation level was found in *in vivo* matured oocytes compared to *in vitro* matured ones in our RRBS study (Jiang et al., 2018), which only detected the clustered CGs that are mainly located within CpG islands (CGI). However, only a minor increase in DNA methylation level was observed after *in vivo* maturation (Duan et al., 2019), which is consistent with mouse studies (Kono et al., 1996; Smallwood et al., 2011) and most recently found in humans as well (Ye et al., 2020). A total of 801 DMRs associated with 68 genes, were found differentially methylated between *in vivo* and *in vitro* matured oocytes. Interestingly, many of these have not been characterized for their roles in maturation, making them good candidates for gene-specific epigenetic modification studies (Duan et al., 2019). These observations provide the underlying mechanism for the abnormal gene expression and reduced embryo and fetal development when oocytes are matured *in vitro*. Interestingly, imprinted loci (e.g., *H19/IGF2, PEG3, SNRPN*), in which epigenetic aberrations are commonly found in imprinting defects in human, mouse and bovine embryos, showed no or only minor methylation alteration between *in vivo* and *in vitro* matured bovine oocytes. However, their mRNA expression levels were changed when matured *in vitro* (Duan et al., 2019), indicating that regulatory mechanisms other than DNA methylation may affect these loci and subsequent oocyte competence and developmental potential of embryos (Heinzmann et al., 2011).

IVM culture conditions influence oocyte competence. For example, when a defined maturation medium with three cytokines (FGF2, LIF, and IGF1) dubbed “FLI medium,” was used, researchers saw improved nuclear maturation of oocytes derived from immature porcine ovaries and a significant increase in blastocyst rate (Yuan et al., 2017). The introduction of simulated physiological oocyte maturation (SPOM) has substantially improved bovine embryo development *in vitro* (Albuz et al., 2010). Many factors in the culture medium have been identified to be involved in epigenetic signature alteration of *in vitro* matured oocytes, including non-esterified fatty acids (NEFA) (Desmet et al., 2016). In contrast to the changes in DNA methylation during *in vitro* maturation that we previously discussed, histone modifications have shown more dynamic changes. H3K9me2, which is strongly associated with transcription repression and acts as a DNA methylation protector (Nakamura et al., 2012), is present from the GV stage until the end of the maturation period, while H4K12ac, which is associated with active promoters, declines drastically after the break down of the germinal vesicle (Racedo et al., 2009). Unlike DNA methylation, genome-wide histone modification dynamics are not available during *in vitro* maturation, thus ChIP-Seq analyses are necessary to understand the effects of this process.

Oocyte cryopreservation is another routine procedure in IVP. It has been established that vitrification affects cellular structures of oocytes and the developmental potential of embryos (Khalili et al., 2017). Chen H. et al. (2016) showed that DNA methylation and H3K9me3 levels are reduced in the bovine oocytes after vitrification, leading to a drastic decline in blastocyst rate. Given the fact that DNA methylation and H3K9me3 are usually associated with gene silencing and heterochromatin formation (Becker et al., 2016), lower abundance of these two epigenetic modifications indicates a permissive and more relaxed chromatin state. This may lead to aberrant epigenetic regulation and expression of imprinted genes (Chen H. et al., 2016). In another study evaluating the effect of different freezing protocols on DNA methylation levels of bovine oocytes, opposing results were obtained depending on the method, with decreased global DNA methylation in slow freezing and DMSO groups, and elevated DNA methylation with the propylene glycol protocol (Hu et al., 2012). Advanced sequencing-based methods will provide more locus-specific information, and are therefore more instructive in directing future studies and possible protocol modifications.

### Epigenetic Modifications Associated With Sperm Fertility in Livestock Species

Male fertility is critical for livestock reproduction, and is mostly influenced by environmental, management and epigenetic factors. There is a growing interest to develop potent epigenomic biomarkers for bull fertility via a systematic approach by compiling epigenomic datasets and associations with reliable phenotypic data.

It has been proposed that sperm-borne factors (proteins, mRNAs, DNA methylation, histone modification, and miRNAs) are associated with fertility and are indispensable for early embryo development (Ostermeier et al., 2004; Yuan et al., 2016). The current knowledge in humans about the different epigenetic signals in sperm that are responsive to environment and evidence of sperm-borne epigenetic factors is well-reviewed in Donkin and Barres (2018). In cattle, studies using sperm samples from low and high fertility bulls have suggested various sperm factors associated with bull fertility, including sperm proteomics, gene expression, sperm protamine status, macromolecules, metabolomics, superoxide dismutase, and amino acids of seminal plasma (Peddinti et al., 2008; Feugang et al., 2010; Govindaraju et al., 2012a; De Oliveira et al., 2013; Dogan et al., 2015; Grant et al., 2015; Kaya and Memili, 2016; Kutchy et al., 2017, 2019; Velho et al., 2018, 2019; Viana et al., 2018; Menezes et al., 2019; Ugur et al., 2019; Memili et al., 2020).

Epigenetic modifications are reported to be associated with sperm fertility, although the mechanisms governing this process remain unclear. For example, acetylation and methylation of H3K27 (H3K27ac and H3K27me3) in sperm has been correlated with bull fertility (Kutchy et al., 2018). Furthermore, inadequate histone replacement in sperm coincides with reduced fertility in bulls (Dogan et al., 2015). Genome-wide H3K4me2 and H3K27me3 profiles were also analyzed in the sperm of water buffalo bulls with divergent fertility. When comparing buffalo sperm from bulls with high fertility and sub-fertility, a total of 84 genes associated with H3K4me2 and 80 genes associated with H3K27me3 were differentially enriched (Verma et al., 2015).

MicroRNAs can also influence male fertility by regulating gene expression (Govindaraju et al., 2012b). Sperm miR-15a and miR-29b have been reported to be associated with bull fertility (Menezes et al., 2020). When researchers looked at small RNAs in sperm with low and high motility from a single bull by RNA-sequencing, they found altered miRNA and piRNA expression (Capra et al., 2017). The characterization of the epigenome in high and low motility bovine sperm also had methylation variations that affected genes involved chromatin organization (Capra et al., 2019). Profiles of the bull sperm small non-coding RNAs across breeds was most recently characterized and showed that miRNAs make up about 20% of the RNA population. This study also increased the list of known miRNAs in bovine sperm considerably (Sellem et al., 2020).

Recent studies using RRBS and WGBS have fully characterized the bull sperm methylome and identified partially methylated domains and hypomethylated regions unique to sperm when compared to somatic tissues (Perrier et al., 2018; Zhou et al., 2018, 2020; Fang et al., 2019). Additionally, assessment of the DNA methylation of spermatozoa between high and low fertility bulls revealed 76 DMRs (Kropp et al., 2017). These studies provide initial steps toward understanding the roles of small non-coding RNAs and methylation in sperm and fertility.

Due to the inability to conduct *in vitro* spermatogenesis in livestock species, sperm usually experience less environmental stressors than oocytes. For example, stresses during IVM, including oxygen tension, temperature fluctuation, media composition and osmotic stress, could cause epigenetic alternations in oocytes (El Hajj and Haaf, 2013; Osman et al., 2018). In domestic species, since the establishment of epigenetic modifications has finished before sperm are retrieved and treated for IVP, the chance is much higher that epigenetic defects in the sperm genome come from perturbation during *in vivo* spermatogenesis rather than sperm handling procedures (Urrego et al., 2014). However, adverse conditions may be present in the testis and lead to male infertility by disrupting epigenetic regulation as has been observed in humans (Rajanahally et al., 2019; Sadler-Riggleman et al., 2019). Reactive oxygen species (ROS)-induced oxidative stress is known to cause DNA damage in sperm in mammals (Schieber and Chandel, 2014). A recent study indicates that ROS can also affect the epigenetic reprogramming of sperm after fertilization (Wyck et al., 2018). The AID-TDG mediated base excision repair (BER) pathway has been found to be pivotal for active demethylation of the paternal genome (Kohli and Zhang, 2013). However, in the presence of a DNA lesion, XRCC1, a protein involved in the last step of BER, is recruited to repair the damaged DNA instead of functioning in demethylation; this led to aberrant active demethylation in the male pronucleus of bovine zygotes (Wyck et al., 2018).

### Epigenetics and Embryo Competence

ARTs are widely used to treat human infertility and improve animal production (Sjunnesson, 2019). IVP embryos are also widely used for research and have increasingly become sources of blastocyst transfer in cattle. Moreover, the production of cloned, transgenic and genome-edited animals relies on the *in vitro* production of embryos. More importantly, *in vitro* production of embryos has allowed the elucidation of many important biochemical and molecular processes that occur throughout oocyte maturation, fertilization, and at the different stages of preimplantation embryo development. There is concern that ARTs contribute to developmental failure and long-term epigenetic alterations in the offspring. Environmental perturbations experienced during *in vitro* embryo production can lead to imprinting diseases in humans (Sutcliffe et al., 2006) and large offspring syndrome (LOS) in ruminants (Young et al., 1998; Chen et al., 2013). The underlying mechanisms are largely unknown at present, but alterations in gene expression and epigenetic modifications, largely DNA methylation, during this critical period are thought to be involved in LOS (Wrenzycki and Niemann, 2003; Li et al., 2005; Sutcliffe et al., 2006; Fernandez-Gonzalez et al., 2010). Epigenetic reprogramming also can occur aberrantly in cloned (SCNT) embryos and the incomplete reprogramming of the differentiated somatic cell DNA may contribute to the low efficiency of cloning (Dean et al., 2001). Abnormal epigenetic regulation in cloned embryos has also been discussed (Yang et al., 2007). A comprehensive study comparing both global gene expression patterns and the epigenome of IVP, SCNT-derived livestock embryos to *in vivo* counterparts would be very interesting.

A number of studies have reported that the *in vitro* environment during IVM, IVP and SCNT significantly alters DNA methylation in the embryos in a locus-specific manner (Bourc'his et al., 2001; Dean et al., 2001; Kang et al., 2002; Han et al., 2003; Niemann et al., 2010; Reis e Silva et al., 2012; Smith et al., 2015; Sirard, 2017; Zhao et al., 2020). In bovine SCNT blastocysts, methylation analysis of 41 amplicons associated with 25 developmentally important genes on 15 different chromosomes (a total of 1,079 CpG sites) showed reduced levels of methylation (Niemann et al., 2010). Embryos derived by IVF and SCNT show epigenetic anomalies in DMRs controlling the expression of some imprinted genes, including *SNRPN, H19/IGF2*, and *IGF2R* (Smith et al., 2015). In another study using immunostaining analysis, DNA methylation reprogramming in bovine preimplantation embryos from SCNT and IVF was compared. The results showed that global DNA methylation followed a typical pattern of demethylation and remethylation in IVF preimplantation embryos; however, the genome remained hypermethylated in SCNT preimplantation embryos (Zhang et al., 2016a). Interestingly, the aberrant methylation of satellite 1 regions in bovine SCNT embryos was corrected as embryo development and differentiation takes places (Sawai et al., 2010). By using EmbryoGENE DNA Methylation Array, Salilew-Wondim et al. identified altered DNA methylation in blastocysts collected *in vivo* after being subjected to various time periods of *in vitro* culture, clearly indicating that *in vitro* culture conditions perturb the epigenetic profiles of preimplantation embryos (Salilew-Wondim et al., 2015, 2018). Most recently, the WGBS technique was used to analyze the methylation patterns of bovine blastocysts derived from *in vivo*, IVF, or IVF with vitrified oocytes and revealed a large number of DMRs that may contribute to the differences in quality between *in vitro* and *in vivo* derived embryos (Zhao et al., 2020). In porcine embryos, the DNA methylation level was increased in *in vitro* embryos compared to *in vivo* ones suggesting the adverse effect of *in vitro* culture on the DNA methylome (Deshmukh et al., 2011). SCNT embryos also had higher DNA methylation levels compared to their fertilized counterparts in sheep (Beaujean et al., 2004). In sheep LOS models, reduced expression of *IGF2R* but not *IGF2* [whose overexpression due to loss of imprinting status is postulated in human fetal overgrowth syndromes (Reik and Maher, 1997)] was caused by loss of methylation on the second intron DMR (Young et al., 2001).

Histone modifications, specifically histone methylation, was also shown to be a key epigenetic barrier to epigenetic reprogramming in SCNT embryos (Matoba et al., 2014). In bovine, immunofluorescence analysis revealed that the acetylation and methylation levels of H3K9ac, H3K18ac, H4K5ac, H4K8ac, H3K4me3, and H3K9me2 were abnormally increased in the SCNT embryos from the zygote to the 8-cell stage, while H4K8ac and H4K5ac were abnormal when compared with the IVF controls. After EGA, the distribution and the pattern of the histone modifications were similar in both SCNT and IVF embryos (Wu et al., 2011). The hypermethylation of H3K9 was also observed in SCNT embryos and associated with DNA hypermethylation; in addition, an association between the epigenetic modifications and the developmental potential of cloned embryos was established (Santos et al., 2003). In porcine, global hypermethylation of H3K27me3 was observed in early cloned embryos compared with IVF embryos (Xie et al., 2016). Cao et al. (2015) compared multiple histone methylation modifications, including transcriptionally repressive (H3K9me2, H3K9me3, H3K27me2, H3K27me3, H4K20me2, and H4K20me3) and active modifications (H3K4me2, H3K4me3, H3K36me2, H3K36me3, H3K79me2, and H3K79me3) between porcine SCNT and IVF embryos at different developmental stages. Histone methylation exhibited stage-specific abnormal patterns in SCNT embryos (Cao et al., 2015). IVP may also change the localization of histone PTMs systematically, separate from SCNT. Between *in vivo* and *in vitro* derived bovine embryos, histone modifying enzymes (e.g., PRMT5, KDM5B, KAT8, HDAC1, and HAT1, etc.) were differentially expressed during preimplantation development (Duan et al., 2019). It has been theorized that the low efficiency of SCNT embryo development rate results, in part, from the divergent DNA methylation and histone modification status between terminally differentiated somatic cells and mature spermatozoa (Peat and Reik, 2012; Jin et al., 2017).

A number of studies have defined precise approaches to remove epigenetic barriers and improve the efficiency of SCNT. In the mouse, treatment with histone deacetylase inhibitors (Kishigami et al., 2006, 2007; Van Thuan et al., 2009), the overexpression of H3K9me3 demethylase *Kdm4b/4d* (Matoba et al., 2014; Liu W. et al., 2016), the correction of abnormal DNA remethylation (Gao et al., 2018) or the deletion of *Xist* on the active X chromosome (Inoue et al., 2010) can significantly improve the developmental potential of SCNT embryos. This significant body of work provides considerable evidence that aberrant epigenetic modifications are the major barriers to complete reprogramming of the donor cell during SCNT.

In porcine, GSK126 and GSK-J4, two small molecule inhibitors of H3K27me3 methylase (EZH2) and demethylases (UTX/JMJD3), were used to improve the developmental efficiency of cloned embryos by reducing the H3K27me3 level (GSK126) (Xie et al., 2016). However, GSK-J4 treatment increased the H3K27me3 level in cloned embryos and decreased the cloned embryo development (Xie et al., 2016). Another study showed that BIX-01294 (a specific inhibitor of histone-lysine methyltransferase of H3K9) enhanced the developmental competence of porcine SCNT embryos (Huang et al., 2016). In addition, use of histone deacetylase inhibitors (e.g., trichostatin A) in the SCNT protocol has been shown to improve both *in vitro* and *in vivo* developmental competence in pigs (Zhao et al., 2010). In bovine, it has been reported that inhibiting H3K9 methyltransferases (SUV39H1) or injecting H3K9 demethylases (KDM4E) in SCNT embryos could improve blastocyst rates similar to what was observed in mice (Zhang et al., 2017; Liu et al., 2018).

Establishing proper epigenetic modifications during gametogenesis and embryogenesis is an important aspect in reproduction and embryo biotechnology. The reprogramming process may be influenced by external and internal factors that result in improper epigenetic changes in germ cells and embryos. Therefore, a combination of epigenetic and other factors could be responsible for the decreased developmental competence of oocytes matured *in vitro* and/or embryos produced *in vitro*. Further comprehensive epigenetic studies are required to compare embryos produced with different ARTs in a stage specific manner using high-throughput sequencing approaches. Also, a full mechanistic description of the many facets of epigenetic reprogramming during this developmental timeframe is needed. Understanding the epigenetic mechanisms is essential to gain insights into normal molecular regulation and correlate it with unperturbed embryonic and fetal development. This would also help to improve ART success and develop new approaches to improve the fertility of animals.

## The Effects of Parental Environmental Exposure on Epigenetics and Reproduction Phenotypes

Animal models, such as rats and mice, have been used extensively to study correlations between epigenetics and disease and there is emerging research in this area using livestock. Considerable work has been focused on studying developmental origins of adult health and disease since it was hypothesized over 30 years ago (Barker, 1990, 1995). Epidemiological studies in humans have shown that parental malnutrition can affect offspring much later in life and extend into the next generation (Heijmans et al., 2008; Pembrey et al., 2014; Eriksen et al., 2017). As a notable example, mothers who were pregnant during the Nazi imposed food crisis in the Netherlands in 1944, also known as the Dutch Hunger Winter, had children with a wide range of health problems as adults, including type II diabetes, obesity, cancer, cardiovascular disease and schizophrenia (Heijmans et al., 2008). Higher body weight was also observed in the next generation in adulthood (Painter et al., 2008; Veenendaal et al., 2013). Tobi et al. (2014) used reduced representation bisulfite sequencing to examine DNA from whole blood in individuals that experienced the Dutch Hunger Winter *in utero* compared with siblings born before or after the period. They found 181 differentially methylated regions in exposed individuals compared to their siblings. The regions were enriched for genes involved in early development (Tobi et al., 2014). The fact that the authors could detect differences more than 60 years after the event emphasizes the nature of epigenetic mechanisms and the importance of epigenetic reprogramming during early development.

Nutrient intake is one of the most direct influences of environment on phenotype. The interaction between nutrition and reproduction has important implications for fertility, especially in cows (Martin et al., 2010). In cattle, prenatal, early post-natal and juvenile nutritional scenarios impact reproductive activity of females later in life; thus, there is the potential for strategic nutritional management during these critical times to program reproduction (Funston et al., 2010). Nutrition is important for epigenetics. For example, nutrients like folate, choline, b-vitamins (B2, B6, B12), and methionine are methyl donors to *S-*adenosylmethionine (SAM), which is the substrate used by DNA and histone methyltransferases in the one-carbon metabolism pathway, reviewed in Clare et al. (2019). Research in both cattle and sheep has shown that maternal diet (supplemented with methyl-donors) during the peri-conception period and/or pregnancy can alter DNA methylation in embryos and in analyzed fetal tissues (Sinclair et al., 2007; Lan et al., 2013; Acosta et al., 2016).

A lot of the fetal programming work conducted in livestock species has been focused on nutrient restriction during pregnancy and its impacts on reproductive function, behavior and growth, reviewed in Ashworth et al. (2009) and Sinclair et al. (2016). In pigs, studies have found significant effects of maternal malnutrition on offspring gene expression, growth, and metabolism to name a few (Leibfried-Rutledge et al., 1987; Barker, 1990; Li et al., 1993; Gonzalez-Bulnes et al., 2014; Ji et al., 2017; Franczak et al., 2018). For instance, Franczak et al. (2018) found over 450 highly differentially expressed genes (>5 fold and mostly up-regulated) between day 15 and 16 pig embryos from gilts fed a normal control diet and gilts fed a restricted diet during fertilization and early preimplantation development (days 0–9). In sheep and goats, calorie restriction during pregnancy resulted in aberrant imprinted (*IGF2, PHLDA2, DIRS3, SLC22A18*) (Duan et al., 2018) (*IGF2R*) (Williams-Wyss et al., 2014) and epigenetic regulator (*TET1, MBD2*) gene expression, respectively, in the tissues of late gestation fetuses (Li et al., 2018). Nutrient restriction during the peri-conception period in sheep also resulted in reduced methylation and increased H3K9 acetylation in the promoters of the hypothalamic genes proopiomelanocortin and the glucocorticoid receptor in fetuses, indicating the role of maternal malnutrition's programming of metabolic and stress response issues later in life (Stevens et al., 2010; Begum et al., 2012). Very recent work by Toschi et al. (2020) in sheep found that maternal peri-conceptional (−14/+28 days) nutrient restriction can also affect the sperm methylome of offspring. Using RRBS analysis, the authors identified 244 DMRs in the sperm of offspring that experienced poor nutrition *in utero* compared with control rams. They also found reduced sperm motility, abnormal chromatin structure and a reduction in the ability to produce blastocysts in this treatment group. Interestingly, folic acid supplementation during the restriction period also created many DMRs, but this treatment rescued the blastocyst rate (Toschi et al., 2020). This result supports the concept of using management interventions to offset epigenetic perturbations and improve reproductive outcomes.

The previously discussed work outlines research that induced nutrient restriction during peri-conception or pregnancy; however, recent work has focused on understanding the epigenetic impacts of post-partum negative energy balance on oocyte methylation in high-producing dairy cows. O'Doherty et al. (2014) looked at imprinted gene methylation during the early, mid and late post-partum periods and found highly variable methylation levels across the time points for several genes. A follow up IVM experiment found specific effects of non-esterified fatty acids (NEFAs) and NEFAs + SAM on the methylation of *PLAGL1* (O'Doherty et al., 2014). Recent work expanded on this candidate gene analysis and used WGBS to examine the effects of negative energy balance on the epigenome of *in vivo* oocytes collected at early (~37 days post-partum) and mid (~65 days post-partum) time points vs. oocytes from heifers (Poirier et al., 2020). The oocytes collected during the early negative energy balance period displayed significant methylation variation within the group (3 replicate pools of oocytes); they also had lower methylation levels overall compared with the mid post-partum and control oocytes. These two factors combined led to the identification of a vast amount of DMRs unique to the early group, many of which were associated with genes involved in key metabolic processes. It is also of interest that a large majority of the imprinted gene bodies analyzed had higher methylation levels in the early post-partum oocytes compared with the other groups. One region, a uniquely differentially methylated CpG island in the early post-partum group specifically mapped to the imprinted gene, *MEST*. Oocytes collected at the mid post-partum time point were much more similar to control oocytes (in overall methylation level and number of DMRs), indicating a degree of recovery when the cows returned to positive energy status (Poirier et al., 2020). Taken together, this work indicates that nutritional stress can impact the methylome of developing gametes, which likely has impacts for gamete viability and developmental potential.

In humans and rodents, studies have shown that paternal nutritional status is associated with metabolic disruptions in the offspring (Chen et al., 2016a). Malnutrition affects sperm viability in males of various species. In rodents, malnourished fathers produce developmentally delayed and metabolically compromised embryos; the conceptuses also have altered placental development (Carone et al., 2010). The effects of paternal malnutrition on the reproductive physiology of adult offspring have also been observed. In mice, two generations of progeny from fathers who ingested a low-protein diet during the preconception period had abnormalities in their reproductive organs (Carone et al., 2010). In female offspring of malnourished fathers, oocyte meiotic competence was reduced, expression of glucose transporters in ovaries and cumulus cells was altered, and reproductive ability was compromised, as seen by lower fertilization and cleavage rates as well as embryos with delayed development (Ashworth et al., 2009). In cattle production systems, bulls are commonly placed on high-energy rations during development to result in a high rate of weight gain (Allen et al., 2017). Energy expenditure during the breeding season commonly results in weight loss in bulls, especially in bulls that have not reached their mature body weight and are still undergoing post-pubertal reproductive maturation (Cardoso et al., 2014). Undernutrition can also occur in pastures or range-based systems, due to normal seasonal variation in nutrient availability (Hills et al., 2015). Therefore, metabolic scenarios related to weight loss and undernutrition could affect seminal parameters and influence epigenetic transmission of traits. These events support the role of sires in the nutritional programming of reproductive function.

Bull fertility is a critical factor dictating economic potential in cattle production systems. Fertility is affected by several factors, including management, nutrition, disease, stress, age, and genetics. A decline in bull fertility affects the conception rate of herds, resulting in decreased production and therefore decreased profit. The ability to confidently predict male fertility would be a boon to the livestock industry. For example, fertility differences among different breeds and even individual bulls within same breed have been well-documented (Den Daas et al., 1998; Dejarnette, 2005). It has been shown that sperm molecular differences between individuals are associated with specific phenotypes of bull infertility (Dogan et al., 2015). The “omics” approaches, such as genomics, transcriptomics, proteomics, metabolomics and epigenomics, have been used to ascertain molecular determinants of bull fertility (Feugang et al., 2010; De Oliveira et al., 2013; Kumar et al., 2015; Bromfield, 2016; Westfalewicz et al., 2017; Velho et al., 2018; Menezes et al., 2020). For example, recent work using RNA-seq examined sperm-derived RNAs in pre-EGA bovine embryos and found 65 differentially expressed RNAs in embryos fertilized with sperm from bulls with low and high fertility (Gross et al., 2019). Analyses of inter-individual variations in bull sperm DNA methylation found a number of DMRs with significant associations with reproduction traits like sperm motility, further supporting the notion that epigenetic information can be harnessed to improve production (Liu S. et al., 2019). Despite abundant research, vast gaps in the knowledge base exist, including specific functional genomic signatures coupled with epigenetic profiles in different bull breeds, as well as mechanisms for their involvement in fertility.

In the livestock industry, selected sires have greater opportunities for breeding, in comparison to females. A small number of sires may be able to “nutritionally” program reproduction in a significant number of female progeny. Thus, assessing the impact of paternal nutrition on reproductive function and the potential heritability of acquired traits merits investigation as a viable strategy to mitigate problems with infertility and subfertility.

## Transgenerational Epigenetic Inheritance in Livestock Species

Dramatic phenotypic differences have been established and stabilized between livestock breeds. There are two different models of phenotypic polymorphism: (1) differences between breeds where dramatic phenotypic differences exist due to decades of selective breeding; and (2) environmentally induced phenotypic differences, specifically those induced by nutritional status. There is significant evidence in mammals that individuals can acquire environmentally induced epigenetic modifications that can be passed transgenerationally, reviewed in Daxinger and Whitelaw (2012), Heard and Martienssen (2014), Chen et al. (2016b), and Zhang et al. (2019). In the case of transgenerational inheritance, it is the founder individual that experiences the environmentally induced epigenetic perturbation and this is passed down via gametes to subsequent generations that have never experienced direct exposure. For true transgenerational inheritance when a gestating female has faced the perturbing event, the aberrant epigenetic pattern must persist to the third generation (F3) (Jirtle and Skinner, 2007). This is because the embryo or fetus' primordial germ cells or gametes (which will form the F2 generation) will also potentially be exposed during their development. In males and non-pregnant females, epigenetic persistence to the F2 generation is sufficient (Skinner, 2008). Thus, it becomes clear the difficulties involved in conducting strong transgenerational epigenetic inheritance studies in livestock species, specifically the cost and complexities associated with performing a study across several generations in species with long generation intervals.

There has been some multigenerational research in dairy cattle that showed the granddam's prenatal environment influenced milk production of daughters and granddaughters (Gonzalez-Recio et al., 2012; Gudex et al., 2014). However, since the analysis started with pregnant females, the F2 generation was exposed, and so the work cannot be categorized as transgenerational in nature. Nonetheless, a transgenerational study with epigenetic analysis has been done in swine. Braunschweig et al. (2012) examined Swiss Large White F2 animals to identify effects of a methyl-donor enriched diet fed to founder boars. They found differences in carcass traits (the control diet progeny tended to be fatter with a trend of decreased shoulder muscle percentage) and gene expression in the liver (64 DEGs), muscle (79 DEGs), and kidney (53 DEGs) of pigs whose grandsires had the experimental diet vs. pigs that descended from control diet fed pigs. The authors examined the DNA methylation pattern of the promoter region of a few selected genes and found reduced methylation of the promoter for the gene iodotyrosine deiodinase in the livers of the experimental progeny; the methylation level was correlated with gene expression level. While this study is limited by the number of F2 pigs analyzed (8 for each group) and the lack of a genome-wide epigenetic analysis, it is the first attempt to examine transgenerational inheritance in a livestock species (Braunschweig et al., 2012).

There is even compelling evidence for transgenerational inheritance in humans. Among the epidemiological human examples, the detailed analysis of the copious historical data from Överkalix, Sweden demonstrates the effects of famine during pre-pubescence in both girls and boys on subsequent grandchildren's health and longevity (Bygren et al., 2001, 2014; Pembrey et al., 2006; Kaati et al., 2007). Physiological challenges (severe nutrient restriction and stress) to grandparents were linked with health issues in the following generations.

We now appreciate that phenotypic complexity goes beyond Mendelian genetics. It is critical that we continue intensive research in this area to fully characterize the epigenetic (and other) mechanisms involved. Furthermore, despite the difficulties inherent in transgenerational studies, it is imperative to expedite this research in livestock species. It will provide a better understanding of the underlying mechanisms controlling phenotype, animal reproduction and health, and the potential means to induce and/or counteract epigenetic modifications for several generations.

### Searching for Epigenetic Factors That Transmit Acquired Phenotypes

The processes by which environmental information is coded and transmitted inter-generationally via the germline remains unclear. Correlative studies in mammals have suggested that DNA methylation, histone modification and small RNA could contribute to intergenerational inheritance of environment-induced phenotypes. Particularly, evidence from laboratory rodents that these parental environment induced traits could be “memorized” in sperm and transmitted to the future generations, implicating sperm-mediated epigenetic inheritance, reviewed in Chen et al. (2016b) and Zhang et al. (2019). Recently developed technologies in the field of omics including genome-wide high throughput sequencing, dynamic imaging of genomic loci, quantitative proteomics and computational analyses have allowed insight into some of these processes (Gomez et al., 2013; Doherty and Couldrey, 2014; Small et al., 2014).

DNA methylation is a solid candidate for epigenetic inheritance in animals because of its relative stability and our understanding of the mechanisms of its deposition and erasure (Bestor, 2000; Jurkowska et al., 2011). Insights from genome-wide methylome studies suggest that a considerable fraction of the mammalian genome might evade the DNA demethylation that occurs normally during preimplantation and PGC reprogramming (Hackett et al., 2013; Guo et al., 2014; Smith et al., 2014; Tang et al., 2015). These escapee modifications could then be transmitted to future generations. In addition, we know that some regions (e.g., imprinted genes) have to be protected from embryonic epigenetic reprogramming and that perturbations can result in differences in DNA methylation (Barlow and Bartolomei, 2014). It has been shown that sperm DNA methylation is altered by various environmental exposures in mice, and it contributes to transgenerational epigenetic inheritance (Radford et al., 2014). Recent work points toward the possible mechanism for transmitting paternal environmental exposures to the next generation, via sperm DNA methylation, ncRNAs and histone retention (Ben Maamar et al., 2020). Additionally, age-related methylation changes are well-documented in human sperm; more recent studies shed light on this in bulls as well (Lambert et al., 2018; Takeda et al., 2019; Khezri et al., 2020; Wu et al., 2020a). Histone modifications have also been implicated in transgenerational epigenetic inheritance (Siklenka et al., 2015). Several studies have established the histone modification mediating epigenetic memory of the germline in *C. elegans* (Rechtsteiner et al., 2010; Gaydos et al., 2014). This major mechanism of epigenetic inheritance has been intensively reviewed (Skvortsova et al., 2018).

Among the epigenetic mechanisms, sncRNAs appear to play a pivotal role in mediating environmental information transmission through sperm in mammals, reviewed in Chen et al. (2016b) and Zhang et al. (2019). Several sncRNAs can be altered in the spermatozoa from obese and/or diabetic men, male mice and rats. Altered sperm sncRNAs, such as miRNA and tsRNA, have been observed following paternal exposure to diet change or stress (Fullston et al., 2013; Gapp et al., 2014; Chen et al., 2016a; Zhang et al., 2018). Injection of sperm RNA from exposed males (e.g., unhealthy diet, mental stress) has been demonstrated to efficiently induce transgenerational inheritance in mammals (Rassoulzadegan et al., 2006; Wagner et al., 2008; Grandjean et al., 2009; Rodgers et al., 2015; Chen et al., 2016a), suggesting sperm RNA is an active epigenetic modulator of offspring phenotypes. In addition, several RNA modifications such as m^5^C, m^2^G have been found to be enriched in the sperm tsRNA and are sensitive to diet changes (Chen et al., 2016a; Zhang et al., 2018). These sperm RNA modifications could alter RNA structure and thus the interactions between RNA, DNA and proteins, leading to potentially greater consequences and wide-ranging effects (Zhang et al., 2018). Wu et al. (2020b) identified distinct differences in sperm-borne miRNAs from bulls of different ages, several of which targeted genes expressed in early embryos. This newly appreciated role of the sperm as RNA-based carriers of hereditary information provides a promising angle with which to understand aging and the etiology of environmentally induced disease beyond the initial exposure.

While epigenetic information can certainly be transmitted between generations, there is a shortage of research in livestock species thus far. A significant challenge to be met in this research area will be to track epigenetic information from one generation to another in cattle, pigs, sheep and goats. Given the effects of environment and aging on epigenetic modifications, future studies need to be carefully designed. This type of research will be made increasingly possible with the continued complete characterization of epigenetics during livestock early embryo development.

### Potential Application of Epigenetic Information in Livestock Production

Aside from understanding the nature of the epigenetic code with regard to programming offspring phenotypes, perhaps an equally important mission is to control offspring health and production by harnessing this epigenetic information.

First, it is possible that a reliable epigenetic “code” exists, which if interpreted, may allow us to predict future phenotypes with accuracy; this prospect is very exciting. This has obvious implications for male reproduction. For example, bulls can breed a large number of cows each breeding season, and this relatively large reproductive opportunity amplifies the impact of environmental scenarios in a few individuals on the future performance of many progeny. If artificial insemination or other ARTs are used, the potential to acquire abnormal phenotypes due to age or nutrition stress in the sire, for example, can be compounded with the perturbations caused by the ART, thus elevating the spread of the undesirable epigenetic modifications in the offspring. The ability to counteract these effects would be very beneficial to producers and researchers.

Second, epigenetic codes could be used as clinical biomarkers of gamete and embryo viability. Improvement of pregnancy rates with *in vitro* produced embryos is a critical problem that needs to be addressed in economically valuable livestock species. The efficiency of producing viable embryos and the development of such embryos after transferring them to recipients is inferior to their *in vivo* derived counterparts, especially in cattle (Thompson, 1997; Diskin and Morris, 2008; Diskin et al., 2011, 2016). In addition, the offspring can have a high incidence of abnormalities, including large offspring syndrome (LOS), severe placental abnormalities, respiratory problems, prolonged gestation, and dystocia (Young et al., 1998; Yang et al., 2007). Future studies are needed to fully elucidate how epigenetics contributes to these abnormalities, which would therefore help us develop new approaches to improve ARTs and more closely mimic *in vivo* profiles. This would improve gamete quality and embryo viability.

Third, phenotypic polymorphism between cattle breeds may be reflected in the gametes as epigenetic codes and represent a potential opportunity to introgress phenotypic differences from one breed to another by “epigenetic engineering.” Recently genome editing tools, such as CRISPR-Cas9 or TALENs, have been utilized to introduce an allele from one breed into the genome of a dramatically different breed. For example, the polled allele from the Angus breed was edited into the Holstein genome resulting in a naturally polled Holstein (Carlson et al., 2016; Young et al., 2020). Additionally, an allelic variation for the long version of the prolactin receptor (the “slick” allele) from the Senepol breed (heat tolerant breed) was introduced into the Angus breed (meat producing breed) using genome editing technology (Bastiaansen et al., 2018). Like genome editing, there is also the power of epigenome editing. Here, a deactivated Cas9 protein can be used to haul epigenetic modifier cargo (such as DNMT3a, TET3, KDM6B, HAT, etc.) to targeted regions in the genome, turning genes on or off as desired (Gomez et al., 2019).

Fourth, livestock species offer clear advantages to study epigenetic inheritance. For example, the use of the livestock will allow us to readily profile semen parameters such as sperm concentration, total sperm per ejaculate and progressive motility, as well as epigenetic modifications in the sperm in a consecutive manner in each animal, although the heterogeneity of the sperm epigenome would need to be examined. This would represent an advantage over rodent models and may represent a better model for understanding the dynamic changes in humans.

## Concluding Remarks

Our understanding of the different layers of epigenomic regulation during gametogenesis and embryogenesis in livestock species is incomplete. Advances in low-input genomic and epigenomic sequencing technologies are now driving the global comprehensive profiling of germline and embryonic epigenomes, thereby improving our understanding of epigenetic regulation in normal development and assessing the impacts of ARTs and other environmental stressors on gametes and embryos. Also, a more complete understanding of inter- and transgenerational epigenetic inheritance in mammals will encourage further research into how experiences can be encoded by epigenetics; this knowledge can lead to application in livestock production and health. Finally, the development of precise editing or modulation of germline and embryonic epigenomes will enable the design of strategies to counteract adverse conditions or to program production performance in livestock. Such strategies for epigenetically programming performance would constitute a significant change with the potential for long-ranging effects in the livestock industry.

## Author Contributions

All authors listed have made a substantial, direct and intellectual contribution to the work, and approved it for publication.

## Conflict of Interest

The authors declare that the research was conducted in the absence of any commercial or financial relationships that could be construed as a potential conflict of interest.
